# Resignation and Nomination

**Published:** 1857-03

**Authors:** 


### RESIGNATION AND NOMINATION.

It will be seen by reference to the proceedings of the College Association, that Dr. H. R. Smith has resigned the chair of Mechanical Dentistry in the Ohio College of Dental Surgery. For four years Dr. S. has held this chair, performing its arduous duties at a heavy sacrifice of time and health. We hope in his retirement he will meet with renewed health and vigor, and that his labors will be remunerated as his devotion to the interests of the profession deserves.

It will also be seen that Joseph Richardson, D. D. S., M. D., of Cincinnati, is nominated to fill the vacancy occasioned by the resignation of Dr. Smith. Dr. R. is already well known to our readers, and needs no introduction, and if he did, the able article at the beginning of the present number would be sufficient for the purpose.

While losing the services of Prof. Smith in the College, we are gratified to know that, if health permits, we will still enjoy the advantages of his *teaching* in the pages of the Register—that he will still be, as he has been, a regular and popular contributor.

Since writing the above, the Trustees have accepted the resignation of Dr. Smith, and confirmed the nomination of Dr. Richardson, so that the Faculty is again full.

